# A Comparison of Pre- and Post-Treatment Cranial MRI Characteristics in Patients with Pediatric Epilepsy Receiving Levetiracetam

**DOI:** 10.3390/medicina60081355

**Published:** 2024-08-20

**Authors:** Hilal Aydin, Adil Aytac, Erdogan Bulbul, Bahar Yanik, Oguzhan Korkut, Burak Gulcen

**Affiliations:** 1 Department of Pediatrics, Faculty of Medicine, Balikesir University, Balikesir 10145, Türkiye; 2 Department of Radiology, Faculty of Medicine, Balikesir University, Balikesir 10145, Türkiye; dradilaytac@gmail.com (A.A.); drerdoganbulbul@yahoo.com (E.B.); yanbah@yahoo.com (B.Y.); 3 Department of Medical Pharmacology, Faculty of Medicine, Balikesir University, Balikesir 10145, Türkiye; ogkorkut@hotmail.com; 4 Department of Anatomy, Faculty of Medicine, Balikesir University, Balikesir 10145, Türkiye; burakgulcen@gmail.com

**Keywords:** children, epilepsy, pretreatment, post treatment, levetiracetam, cranial magnetic resonance imaging

## Abstract

*Background and Objectives*: This study was performed for the purpose of assessing whether antiepileptic levetiracetam treatment produces a change in brain volumes in children with epilepsy. To that end, we compared the volumes of the basal ganglia (caudate nucleus, putamen, globus, hip-pocampus, and thalamus) at magnetic resonance imaging (MRI) before and after treatment (months 18–24) in pediatric epilepsy patients using levetiracetam. *Materials and Methods*: This retrospective study involved a volumetric comparison of patients presenting to the Balikesir University Medical Faculty pediatric neurology clinic between 01.08.2019 and 01.11.2023 and diagnosed with epilepsy, and who underwent cranial MRI before and 18–24 months after treatment at the radiology department. The demographic and clinical characteristics (age, sex, family history of epilepsy, type of epilepsy, and EEG features (normal, abnormal, epileptiform)) of the patients included in the study were recorded. *Results:* The comparison of basal ganglia volumes at cranial MRI before and at months 18–24 of treatment revealed significant differences in the left caudate nucleus, right putamen, left putamen, left globus pallidus, right thalamus, left thalamus, and right hippocampal regions. *Conclusions:* In conclusion, differing findings are encountered at cranial imaging in patients with epilepsy, depending on the seizure frequency, activity, and the type of antiepileptic drugs used. This study compared basal ganglia volumes on cranial MRIs taken before and 18–24 months after treatment in pediatric epilepsy patients using levetiracetam. A significant increase was observed in the volumes of basal ganglia (caudate nucleus, putamen, globus pallidus, hippocampus, and thalamus) on the MRIs of pediatric epilepsy patients using levetiracetam.

## 1. Introduction

Epilepsy is a neurological disease widely seen in childhood. It consists of the presence of at least two seizures, occurring with no clear cause, more than 24 h apart. Reflex seizures are also included in the definition. Epilepsy can additionally be diagnosed with a single seizure (or reflex seizure) developing with no trigger; in this case, the risk of seizure recurrence with 10 years following a single seizure is the same as that of recurrence following two seizures (60%). A single seizure can be considered sufficient for a diagnosis of epilepsy if consistent with a specific epileptic syndrome [1].

Magnetic resonance imaging (MRI) is the most specific and sensitive of all structural imaging modalities for detecting subtle abnormalities in the evaluation of epilepsy cases. Its essential role is to identify structural abnormalities underlying the etiology of the seizure (tumors, cortical development malformations, hippocampal sclerosis, neurocutaneous diseases, vascular malformations, traumatic lesions, paralyses, residual lesions, etc.), and to assist with the etiological diagnosis and classification of different epilepsies and epileptic syndromes. It is thus also important in terms of establishing accurate prognosis [2,3].

Anatomically, the basal ganglia are deep symmetrical structures of the gray matter. Most authors consider the following structures to be part of the basal ganglia: the caudate and lentiform nuclei, thalamus, subthalamic nuclei, and substantia nigra. The lentiform nucleus consists of the inner globus pallidus and outer putamen (the putamen is part of the structure of the striatum, together with a distinct caudate nucleus) [4].

The basal ganglia are composed of a plethora of pathways, utilizing GABA, dopamine, acetylcholine, and glutamine. There are multiple pathways within the basal ganglia with both excitatory and inhibitory functions. Input to the basal ganglia is received from both the cerebral cortex and the thalamus. Since the cerebral cortex acts as both the primary input center and the ultimate output target, taking information from the cortex, modifying it, and then passing it back to the cortex is regarded as a function of the basal ganglia [5,6].

Levetiracetam is an antiepileptic used in the treatment of focal, myoclonic, and tonic–clonic seizures. The mechanism involved in the antiepileptic effects of levetiracetam is still not fully understood. However, it is thought to be associated with its binding to synaptic vesicle protein 2A (SV2A). This protein forms part of the secretory vesicle membranes that mediate calcium-dependent vesicular neurotransmitter release and is found in all regions of the brain, particularly subcortical areas such as the ganglia and thalamus [7,8].

Epileptic children have been investigated in numerous studies in the literature, although the number addressing the effect of age at onset of epilepsy, seizure groups and frequency, and antiepileptic drug use on brain volumes is much smaller [9,10,11]. However, these few studies have reported significantly smaller brain volumes in epileptic children compared to healthy individuals [9,12].

This study was performed for the purpose of assessing whether antiepileptic levetiracetam treatment produces a change in brain volumes in children with epilepsy. To that end, we compared the volumes of the basal ganglia (caudate nucleus, putamen, globus, hippocampus, and thalamus) at MRI before and after treatment (months 18–24) in pediatric epilepsy patients using levetiracetam.

## 2. Materials and Methods

This retrospective study involved a volumetric comparison of patients presenting to the Balıkesir University Medical Faculty pediatric neurology clinic between 1 August 2019 and 1 November 2023 and diagnosed with epilepsy, and who underwent cranial MRI before and 18–24 months after treatment at the radiology department.

**Figure 1:**
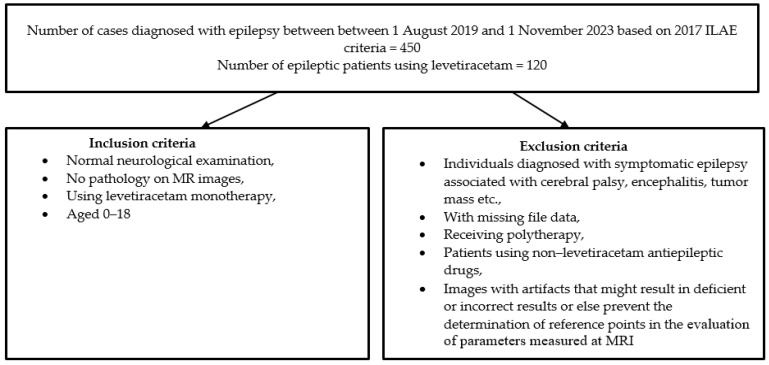
The inclusion and exclusion criteria of the patients enrolled in the study.

Children aged 0–18 years diagnosed with epilepsy by a pediatric neurologist based, with normal neurological examinations, with no macroscopic pathology at MRI, and started on levetiracetam therapy only were included in the study. Patients diagnosed with symptomatic epilepsy associated with cerebral palsy, encephalitis, tumor mass, etc., with missing file data, receiving polytherapy using a non-levetiracetam single antiepileptic drug, and patients diagnosed with symptomatic epilepsy associated with cerebral palsy, encephalitis, tumor mass, etc., with missing file data, receiving polytherapy, using single antiepileptic drugs other than levetiracetam, or with artifacts that might result in deficient or incorrect results or else prevent the determination of reference points in the evaluation of parameters measured at MRI were excluded from the study. The inclusion and exclusion criteria are shown in Figure 1.

The demographic and clinical characteristics (age, sex, family history of epilepsy, type of epilepsy, and EEG features (normal, abnormal, epileptiform)) of the patients included in the study were recorded.

Permission for the research was obtained from the Balikesir University Clinical Trials Ethical Committee before commencement (decision no. 2023/166 dated 22 November 2023).

### 2.1. MR Imaging Procedures and Image Analysis

All patients underwent MRI at the time of diagnosis based on a standardized pediatric epilepsy protocol using a 1.5-T MR system (Ingenia, release 5.3–5.7 software, Philips Medical Systems, Best, The Netherlands). The protocol consisted of axial T1-weighted imaging (WI) spin-echo (SE)(repetition time/echo time (TR/TE): 450/15; field of view (FOV): 230 mm, slice thickness: 5 mm, matrix: 308 × 183), axial fat suppressed (FS) T1-WI SE(TR/TE: 633/15; field of view (FOV): 230 mm, slice thickness: 5 mm, matrix: 308 × 183), axial T2-WI turbo spin-echo (TSE) (TR/TE: 5240/100, FOV: 230 mm, slice thickness: 5 mm, matrix: 384 × 237), coronal FS fluid-attenuated inversion recovery (FLAIR) sequence (TR/TE: 11,000/130; FOV: 230 mm, slice thickness: 5 mm, matrix: 256 × 157), coronal T2-WI TSE (TR/TE: 3027/100; FO): 200 mm, slice thickness: 3 mm, matrix: 336 × 217), coronal T1-WI inversion recovery (IR) (TR/TE: 3079/15; field of view (FOV): 200 mm, slice thickness: 3.5 mm, matrix: 336 × 211), coronal FLAIR sequence (TR/TE: 11,000/130; FOV: 230 mm, slice thickness: 3 mm, matrix: 256 × 157), 3D FLAIR sequence (TR/TE4800/315; FOV 250 mm, slice thickness: 1.04 mm, matrix: 216 × 218), and axial plane diffusion-weighted imaging (DWI) (b values of 0 and 1000 s/mm^2^, FOV: 230 mm, slice thickness: 5 mm, matrix 152 × 106). No contrast agents were employed in any case. The images obtained were transferred to a personal computer workstation, after which volumetric analysis was carried out using 3D Slicer (version 4.11.0) software. This permits reliable analysis of the morphometry of structures of interest by means of either manual or semi-automated tracing. Volumetric analysis of the basal ganglia was performed using 3D FLAIR images. Slicer basal ganglia segmentations were produced by means of a thresholding tool. Use of manual tools such as painting and erasing (semi-automatic segmentation) was kept at minimal levels. The segmentations were carried out by (EB) and (AA). The volume of each region of interest was determined automatically as the product of the trace area of each slice and the slice thickness. All volume values were calculated in cm^3^. The segmentation workflow in 3D Slicer (version 4.11.0) software consists of two parts, initialization and adjustment of the segmentation result. Five steps need to be performed in order to initiate segmentation: input volume, input seeds, output volume, target segmented volume, and output label value. The segmented volume selection range, output segmentation volume, and interactive volume rendering of the segmentation results are then performed to adjust the segmentation result. The borders of the caudate nucleus, putamen, globus pallidus, thalamus, and hippocampus are determined by marking their borders on axial, sagittal, and coronal images in visually traceable consecutive sections. The segmented volume selection range makes it possible to specify the range of value in the basal ganglia, thalamus, and hippocampus. The output segmentation volume makes it possible to scroll smoothly through the volume values within the selected range. The inclusion of areas outside the targeted anatomical structures into the segmentation volume is thus minimized as much as possible.

### 2.2. Statistical Analysis

Demographic and clinical data were retrieved from the medical records. Data analysis was carried out on SPSS software v.22.0 (SPSS Inc., Chicago, IL, USA). Summary statistics included mean, standard deviation (SD) maximum, and minimum values for continuous variables and percentage and frequency values for categorical variables. After the data had been evaluated for conformity with normal distribution using the Shapiro–Wilk test, statistical comparisons were performed using the non-parametric Wilcoxon test, with *p* values < 0.05 being considered significant.

## 3. Results

Fourteen patients met the study criteria, three boys (21.4%) and eleven (78.6%) girls with a mean age of 13 ± 2.18 (8–15) years. Nine (64.3%) had experienced focal type seizures and five (35.7%) had experienced the generalized type. A family history of seizures was present in five cases (35.7%). The patients’ clinical characteristics are shown in Table 1.

Comparison of basal ganglia volumes at cranial MRI before and at months 18–24 of treatment revealed significant differences in the left caudate nucleus, right putamen, left putamen, left globus pallidus, right thalamus, left thalamus, and right hippocampal regions (*p* = 0.013, *p* = 0.022, *p* = 0.002, *p* = 0.48, *p* = 0.005, *p* = 0.009, and *p* = 0.048, respectively). A volumetric and statistical comparison of the basal ganglia at cranial MRI before and after treatment in the pediatric epilepsy patients using levetiracetam is shown in Table 2 and Table 3.

## 4. Discussion

Normal brain development during childhood includes age-related structural changes, resulting from the selective elimination of neurons (cortical pruning) and increasing myelination [13]. Longitudinal quantitative MRI investigations of healthy children have also demonstrated age- and region-specific declines in cortical thickness and cerebral gray matter volume, along with a concomitant increase in cerebral white matter volume, directly reflecting this neurodevelopmental process [14,15].

The thalamus is the major structure of the diencephalon as a key sensory and motor relay center with wide reciprocal connections to the cortex, subcortex, and cerebellum [16]. The thalamus changes in size as the brain matures and ages. The size of the thalamus has been studied in utero in children older than four years and also in adults. During fetal life, the thalamus increases in size between 20 and 43 weeks of gestation [17]. There is now also known to be a linear trend in terms of a decrease in thalamic volumes from the ages of four to eighteen when controlling for cranial size [18,19,20]. The thalamus has also been shown to decrease in size and become slightly atrophic during the passage into adulthood and later in life [20,21]. However, one missing component in the observation of age-related changes in the thalamus is the first four years of life. In addition to age, sex has also been reported to affect the size of the thalamus, with female children and adolescents having larger thalami relative to gray matter and brain volumes in studies by Xie et al. and Sowell et al. [18,20]. In older subjects, findings regarding sex differences in thalamic size vary across different studies. Mohammadi et al. reported a slightly larger thalamus in males than females whereas Sullivan et al. observed no gender difference in terms of thalamic volume [18,21,22]. Thalamus volumetric reference volumes by age groups are presented in Table 4 [23]. 

The majority of previous analyses focusing on basal ganglia laterality have revealed rightward asymmetry of the caudate nucleus [24,25,26,27,28] and leftward asymmetry of the putamen [25,27,28,29] and globus pallidus [25,29], identifying differences in volume without a precise evaluation of the normal reference interval of basal ganglia asymmetry. Based on previous studies, age may contribute to a decrease in the raw volume of the basal ganglia. Age-related bilateral atrophy of putamen and globus pallidus volumes in males was described by Gunning-Dixon et al. [30]. The caudate nucleus volume is not associated with age, which aligns with previous findings from Vernaleken et al. [28], but is inconsistent with Abedelahi et al. [27], Gunning-Dixon et al. [30], and Raz et al. [31]. The mechanisms of age-related shrinkage of the basal ganglia are still unknown. One possible explanation may involve synaptic pruning and neuronal selection. Neuronal network optimization is associated with cognitive development [32]. This sex difference may be related to total cerebral volume, sex chromosomes, hormonal and environmental effects, or to a combination thereof. Previous research shows that males have a larger total cerebral volume than females. Some researchers have described specific cortical regions where this difference is particularly pronounced [2,33,34]. Munro et al. suggested that the sexual dimorphism of the basal ganglia can be explained by the dopamine release system [35]. Sex hormone concentrations may also substantially affect the basal ganglia volume because of the high density of estrogen and androgen receptors [36]. This relationship is driven by estrogen that regulates the activity of dopamine-containing fibers originating in the midbrain and terminating in the basal ganglia [37]. Basal ganglion volumetric reference values by age groups are presented in Table 5 [38,39].

Various pathological processes and drugs are known to affect the anatomy of the brain. For example, the hippocampus has increasingly attracted the interest of researchers due to its ability to generate, maintain, and spread epileptic seizures. Although the hippocampal formation is known to be compromised by neuropathological conditions, many underlying mechanisms related to neuronal gain and loss remain obscure [40].

Hakyemez et al. set out to determine the volumetric and morphological lateralization of major lymphatic structures such as the hippocampus, mammillary body, amygdala, and fornix in 42 patients with complex partial seizures deriving from the temporal lobe. Quantitative measurement revealed a larger hippocampus in the control cases compared to the patient group (*p* < 0.001), with unilateral hippocampal volume loss being observed in 88% of these patients and bilateral loss in 13% [41].

Cerebral atrophy is characterized by parenchymal volume loss and the enlargement of intra- and extra-axial cerebrospinal fluid spaces. It can have numerous causes, including metabolic, demyelinating, neurodegenerative, infectious, inflammatory, cerebrovascular, and post-traumatic processes. It may accompany pathologies in the etiology of epilepsy or may be seen at MRI as a complication of neuronal damage caused by a disease independent of the etiology or as a complication of chronic antiseizure medication use [42,43,44].

Sectional MRI studies in child and adult epilepsy patients reveal significant decreases in cerebellar or cerebral volumes in addition to hippocampal or brain stem atrophy [9,45]. Wozniak et al. reported atrophy in gray matter, the brain, cerebellum, brain stem, putamen, thalamus, hippocampus, and nucleus accumbens in the MRIs of pediatric epilepsy patients relative to healthy controls. Those authors concluded that evaluating volumetric changes in brain structures might represent a useful diagnostic tool in children with epilepsy [46]. In a 2016 study, Tondelli et al. compared healthy controls to adolescents with juvenile absence epilepsy (JAE). Those authors detected a decrease in both gray matter volume and surface area in the bilateral frontal regions, anterior cingulate, and right mesial temporal lobe in the JAE cases. Correlation analysis showed that the decrease in the right anterior and posterior central gyrus surface area was related to the duration of the disease [47].

Research has reported that atrophy in epileptic patients may occur as a result of factors such as hypoxia and treatment with antiepileptic drugs [48]. Antiepileptic drugs are known for their potential neurotoxic effects, although the exact mechanism leading to the reduced gray matter volume remains controversial. Animal studies have demonstrated a neurotoxic effect for several such drugs. Perinatal exposure to phenobarbital and phenytoin has been shown to lead to a decrease in brain weight in rats, as well as to a decline in Purkinje cells and granule cells in the cerebellum [49,50]. Microscopic examinations have revealed apoptotic neurodegeneration in the developing rat brain, thus confirming the neurotoxic effect of these drugs in utero. These effects may derive from effects on cell proliferation, migration, synaptogenesis, and synaptic plasticity [51,52,53,54].

Levetiracetam is a second-generation antiepileptic that is relatively better tolerated than other antiepileptic drugs, and one that has become frequently employed by clinicians [55]. Studies have shown that it also exhibits anti-ictogenic, neuroprotective, anti-inflammatory, and antioxidant effects [56]. Levetiracetam plays a role in the regulation of Ca2+-dependent neurotransmitter release by binding to SV2A [57]. SV2A can be found in all regions of the brain, particularly in subcortical regions such as the basal ganglia and thalamus, and studies have also reported that the density of SV2A can change in association with Alzheimer’s and Parkinson’s diseases, frontotemporal dementia, Huntington disease, epilepsy, schizophrenia, HIV infection, obesity, ischemic stroke, and normal aging [8,58]. Epilepsy is associated with increased excitatory neuronal activity. Levetiracetam has been shown to prevent hyperexcitability, exemplified by chronic treatment that fully prevents the development of hippocampal hyperexcitability in a model of pilocarpine-induced status epilepticus [59]. Levetiracetam exhibits an anticonvulsant effect that provides long-term protection against behavioral seizures and neuronal injury in a rat neonatal model of hypoxia-induced seizures [60].

Studies have suggested that levetiracetam can control recurring postoperative seizures better than other antiepileptic drugs [61,62]. Jehi et al. suggested that this may be associated with the anti-epileptogenic characteristic of levetiracetam [63]. The proposed mechanisms involved in this anti-epileptogenic effect in animals range from inhibition of interleukin-1b inflammatory markers in the hippocampus and piriform cortex or transforming growth factor b in astroglia, to modulation of presynaptic P/Q-type voltage-dependent calcium channels in granule cells of the dentate gyrus [64,65,66].

Wandschneider et al. compared patients with temporal lobe epilepsy (TLE) using levetiracetam with those not using it and reported that although recall performances were equal between the two groups, normalization of the left medial temporal lobe deactivation during performance of a verbal task was observed in the patients using levetiracetam. A similar situation was detected in the right hippocampus during a visual–spatial task, additionally becoming more marked as the levetiracetam dosage was increased. This improvement in task-related activation patterns was interpreted as a beneficial effect of the drug [67]. At the same time, those authors concluded that the patients receiving levetiracetam exhibited normalized functional network deactivations in the right temporal lobe in right TLE when performing right-lateralizing visual–spatial tasks and in the left temporal lobe in left TLE when carrying out verbal tasks [67]. In their voxel-based manometry, from the analyses of cranial images from experimental animals receiving intravenous levetiracetam and valproic acid, Tang et al. demonstrated that valproic acid caused an increase in gray matter volume in the right geniculate body and right pulvinar, while levetiracetam caused a decrease in white matter volume in the right parietal lobe, right pulvinar, and right occipital lobe [68].

## 5. Conclusions

In conclusion, differing findings are encountered at cranial imaging in patients with epilepsy, depending on the seizure frequency, activity, and the type of antiepileptic drugs used. This study compared basal ganglia volumes on cranial MRIs taken before and 18–24 months after treatment in pediatric epilepsy patients using levetiracetam. A significant increase was observed in the volumes of basal ganglia (caudate nucleus, putamen, globus pallidus, hippocampus, and thalamus) on the MRIs of pediatric epilepsy patients using levetiracetam. Brain development varies depending on age and sex. Further studies involving larger patient groups are now needed for a clearer understanding of the net effect of levetiracetam in the light of age and gender factors.

Considering that volume can change in line with age and long-term treatments, the presence of a healthy control group is also necessary for a more accurate analysis of the relationship between volume change and the disease and/or medication The principal limitations of this study are the small patient number; the fact that volume changes could not be correlated with the levetiracetam dosage, age group, or epilepsy type; the broad time elapsing between the pre- and post-treatment periods, meaning that the patient’s growth during that time may also potentially have affected those values; the retrospective nature of the research; and finally the absence of a control group. Further prospective studies involving more patients and centers and in which evaluation using different techniques is employed are now needed.

## Figures and Tables

**Table 1:** The patients’ clinical characteristics.

**Age**	13 ± 2.18 (8–15)
**Gender**	
Female	11 (78.6%)
Male	3 (21.4%)
**Type of epilepsy**	
Focal	9 (64.3%)
Generalized	5 (35.7%)
**EEG findings**	
Normal	3 (21.4%)
Abnormal	6 (42.9%)
Epileptiform	5 (35.7%)
**Family history of epilepsy**	5 (35.7%)

**Table 2:** A volumetric comparison of basal ganglia on pre- and post-treatment cranial MRIs from the pediatric epilepsy patients using levetiracetam.

Patient	Pre-Treatment Volume (cm^3^)										Post-Treatment Volume (cm^3^)									
ID	R, CN	L, CN	R, PTM	L, PTM	R, GP	L, GP	R, Th	L, Th	R, HCM	L, HCM	R, CN	L, CN	R, PTM	L, PTM	R, GP	L, GP	R, Th	L, Th	R, HCM	L, HCM
1	3.915	4.012	6.223	6.158	1.289	1.175	4.307	4.234	2.568	2.407	4.017	4.089	6.513	6.491	1.349	1.264	4.442	4.216	2.590	2.447
2	4.703	4.660	6.601	6.811	1.873	1.803	5.003	5.141	2.826	2.904	4.759	4.716	6.903	7.025	1.829	1.859	5.419	5.284	2.773	2.802
3	3.573	3.751	6.169	6.112	1.652	1.492	4.433	4.280	2.502	2.635	3.512	3.552	6.271	6.317	1.711	1.505	4.486	4.429	2.534	2.589
4	4.663	4.690	6.529	6.469	1.903	1.839	5.091	5.524	2.639	2.555	4.672	4.912	6.905	6.996	2.004	1.904	5.088	5.690	2.785	2.690
5	4.126	4.317	6.515	6.258	1.713	1.814	5.142	5.253	2.489	2.557	4.235	4.699	6.445	6.557	1.729	1.956	5.214	5.238	2.875	2.947
6	4.451	4.391	6.251	6.124	1.759	1.741	4.568	4.696	2.711	2.735	4.830	4.721	6.382	6.235	1.779	1.813	4.817	4.794	2.736	2.795
7	3.515	3.485	5.904	5.842	1.413	1.284	4.480	4.655	2.479	2.364	3.603	3.712	6.204	6.312	1.454	1.481	4.518	4.774	2.490	2.412
8	4.729	4.518	6.572	6.719	1.789	1.932	4.572	4.713	2.446	2.561	4.594	4.821	6.610	6.880	1.816	1.953	4.858	4.890	2.411	2.510
9	4.442	4.321	6.148	6.312	1.585	1.502	4.311	4.257	2.616	2.544	4.311	4.382	6.231	6.309	1.615	1.546	4.501	4.315	2.772	2.591
10	4.414	4.566	6.573	6.048	1.376	1.359	4.316	4.119	2.444	2.650	4.741	4.832	6.846	6.370	1.220	1.245	4.650	4.595	2.559	2.616
11	4.886	4.784	6.786	6.317	1.685	1.699	4.913	4.754	2.690	2.557	4.534	4.963	6.800	6.805	1.663	1.612	5.214	5.095	2.901	2.954
12	3.687	3.788	6.217	6.018	1.136	1.184	4.305	4.630	2.475	2.506	3.855	3.961	6.325	6.346	1.289	1.275	4.413	4.527	2.377	2.435
13	4.780	4.874	6.884	6.732	1.873	1.719	4.776	4.890	2.220	2.175	4.528	4.729	6.552	6.718	1.813	1.749	4.635	4.904	2.457	2.519
14	4.269	4.342	6.542	6.438	1.691	1.712	4.693	4.524	2.776	2.684	4.371	4.402	6.623	6.501	1.700	1.721	4.917	4.905	2.777	2.746

R: right, L; left, GP; globus pallidus, Th; thalamus, CN; caudate nucleus, PTM; putamen, HCM; hippocampus.

**Table 3:** A statistical volumetric comparison of basal ganglia on pre- and post-treatment cranial MRIs from the pediatric epilepsy patients using levetiracetam.

	Pre-Treatment Volume (cm^3^) Mean ± SD (min–max)	Post-Treatment Volume (cm^3^) Mean ± SD (min–max)	*p*
R, CN	4.29 ± 0.46 (3.515–4.886)	4.32 ± 0.43 (3.512–4.830)	0.594
L, CN	4.32 ± 0.41 (3.485–4.874)	4.46 ± 0.46 (3.552–4.963)	0.013
R, PTM	6.422 ± 0.27 (5.904–6.884)	6.54 ± 0.24 (6.204–6.905)	0.022
L, PTM	6.31 ± 0.29 (5.842–6.811)	6.56 ± 0.27 (6.235–7.025)	0.02
R, GP	1.62 ± 0.23 (1.136–1.903)	1.64 ± 0.22 (1.220–2.004)	0.331
L, GP	1.58 ± 0.25 (1.175–1.932)	1.63 ± 0.255 (1.245–1.956)	0.048
R, Th	4.63 ± 0.30 (4.305–5.142)	4.97 ± 0.33 (4.413–5.419)	0.005
L, Th	4.69 ± 0.40 (4.11–5.52)	4.83 ± 0.40 (4.216–5.690)	0.009
R, HCM	2.56 ± 0.15 (2.220–2.826)	2.64 ± 0.17 (2.377–2.901)	0.048
L, HCM	2.55 ± 0.17 (2.175–2.904)	2.64 ± 0.18 (2.412–2.954)	0.140

R: right, L; left, GP; globus pallidus, Th; thalamus, CN; caudate nucleus, PTM; putamen, HCM; hippocampus.

**Table 4:** Thalamus volumetric reference volumes by age groups [23].

Age (years)	Thalamus Volume (cm^3^) Median	IQR
1	4.77	1.38
2	4.45	1.21
3	5.19	1.69
4	5.32	0.60
5	5.10	2.01
6	4.78	1.99
7	6.01	0.5
8	5.90	1.78
9	5.51	1.39
10	5.22	1.45
11	5.81	1.43
12	4.78	1.68
13	5.66	0.64
14	5.60	0.75
15	5.69	1.69
16	5.09	0.97
17	5.71	0.87
18	5.13	1.58

**Table 5:** Basal ganglion volumetric reference values by age groups [38,39].

Age Group (years)	7–9	10–12	13–15	16–18
Right caudate nucleus	4.25 ± 0.58	4.25 ± 0.53	4.26 ± 0.58	4.24 ± 0.61
Left caudate nucleus	4.09 ± 0.49	4.03 ± 0.46	4.03 ± 0.51	4.04 ± 0.49
Right putamen	6.48 ± 0.84	6.52 ± 0.77	6.53 ± 0.73	6.27 ± 0.78
Left putamen	6.76 ± 0.84	6.77 ± 0.79	6.75 ± 0.80	6.47 ± 0.85
Right globus pallidus	1.81 ± 0.24	1.81 ± 0.22	1.79 ± 0.22	1.79 ± 0.25
Left globus pallidus	1.98 ± 0.24	1.99 ± 0.27	1.97 ± 0.30	1.90 ± 0.31
Right hippocampus	2.76 ± 0.29	2.75 ± 0.40	2.69 ± 0.27	3.04 ± 0.46
Left hippocampus	2.56 ± 0.27	2.68 ± 0.45	2.50 ± 0.32	2.85 ± 0.48

## Data Availability

Data are contained within the article. The data will be available on request.
